# Dynamic Hip Screw Plate Length in Stable Intertrochanteric Fracture Neck of Femur: A Systematic Review

**DOI:** 10.7759/cureus.23138

**Published:** 2022-03-14

**Authors:** Aditya Soni, Sandeep Munshi, Niranj G Radhamony, Rajiv Nair, Sachith Sreenivasan

**Affiliations:** 1 Trauma and Orthopaedics, Furness General Hospital, University Hospitals of Morecambe Bay NHS Foundation Trust, Barrow-in-Furness, GBR; 2 Trauma and Orthopaedics, Royal Stoke University Hospital, University Hospitals of North Midlands NHS Trust, Stoke-on-Trent, GBR; 3 Neurosurgery, Auckland City Hospital, Auckland, NZL

**Keywords:** dhs biomechanical, neck of femur fracture, stable intertrochanteric fracture, dhs plate length, dynamic hip screw, dhs

## Abstract

There has always been a debate between the use of four and two-hole plate for the fixation of stable intertrochanteric fractures. The choice is usually influenced by the general practice of the particular institution and the surgeon’s preference. While the dynamic hip screw (DHS) is the implant of choice for stable intertrochanteric fractures of the femur, the length of the side plate to be chosen for optimal results has no clear consensus and previous studies regarding the same have been inconclusive. In our systematic review, we aimed to review the evidence available on the selection of the optimal length of the side plate and bridge the glaring gap that exists in the literature.

Our systematic review included a thorough search of databases like PubMed, Embase, MEDLINE, CINAHL, and the Cochrane Library, using the Preferred Reporting for Systematic Reviews and Meta-Analyses (PRISMA) guidelines. We included both clinical and biomechanical studies satisfying our search criteria on two- and four-hole DHS implants.

A total of 4556 results were obtained from the above databases, sorting out led to final 15 studies on the topic. It was found that the two-hole DHS implant was inferior in terms of lab-controlled biomechanical properties, while only having a slight advantage in terms of real-life postoperative blood transfusions and operative time. At the same time, the two-hole plate was similar to the four-hole plate in other clinical parameters.

In this study, the two-hole plate while appearing promising in a few areas did fall short in other aspects like biomechanical studies, and the use should be reserved for cases where a four-hole plate cannot be used until further randomised control trials are carried out.

## Introduction and background

Hip fractures, especially intertrochanteric fractures of the femur, are one of the most common fractures seen in the elderly in the United Kingdom and are the most common cause of admission of these patients to the orthopaedic ward [1]. The National Hip Fracture Database Annual Report published in March 2021 states that the number of hip fractures reported in the United Kingdom in 2020 was 63,284. This number has remained high in the last four decades despite the advancements in the management of osteoporosis and improvements in the physical support system at the community level [2].

Peri trochanteric fractures have been studied very extensively and dynamic hip screw (DHS) has been the unanimous choice of implant for stable intertrochanteric fractures [3,4], while intramedullary nailing is preferred for the unstable and the subtrochanteric types [5]. In addition, the DHS has also been used for the fixation of undisplaced intracapsular fractures.

A four-hole side plate has been used traditionally for almost all fractures requiring DHS, while a two-hole DHS use has been more sporadic [3,4]. Theoretically, a two-hole DHS plate has the advantage of a shorter operative duration, reduced intraoperative blood loss, and decreased operative site morbidity but there are many more parameters that need to be considered before its use becomes widespread. A definite void was also found in the literature comparing the clinical usefulness of both the implants and recommendations on when to use each.

Numerous studies have been done comparing various aspects of two-hole and four-hole DHS, but to the author’s knowledge, a compilation of the important parameters in the form of a systematic review is inadequate. While some aspects like blood loss, hospital stay, failure rates, and biomechanical aspects have been included previously, the interpretation of the data and analysis appears to be done based on incomplete assessment. Other aspects like fracture healing time, radiation exposure, analgesic use, fracture position at healing, and functional results were not looked at. Following this systematic review, the recommendations may bring about a potential change of practice and also pave way for further focused in-depth research on the same.

Methods

Preferred Reporting for Systematic Reviews and Meta-Analyses (PRISMA) guidelines were followed at every step [6]. A literature search of PubMed, EMBASE, MEDLINE, CINAHL, and the Cochrane library databases was done. Single and various combinations of the following keywords were searched: 'dynamic hip screw', 'sliding hip screw', 'compression hip screw', 'DHS', '2 hole', 'two hole', 'two-hole', '4 hole', 'four-hole', 'four hole', 'biomechanical studies', 'cadaveric studies', 'intertrochanteric fractures', 'neck of femur fractures'. The full text was read through for review.

**Figure 1:**
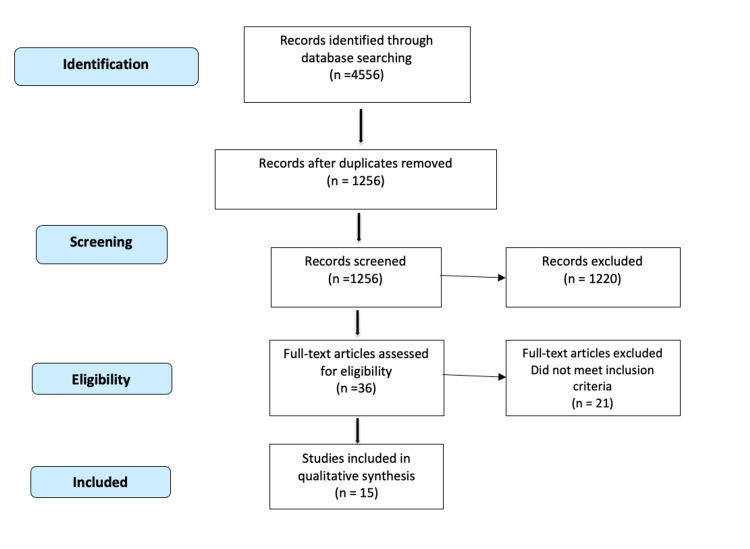
Methodology flow diagram

To manage the risk of bias and maintain quality, selected studies were critically appraised using the Critical Appraisal Skills Programme (CASP) checklist criteria before being included. Papers of all study designs which had a two-hole DHS as the method of fixation in isolation or being compared to a four-hole DHS were included. All cadaveric and artificial bone model studies were actively searched and included to throw light on biomechanical aspects. The literature not written in English and studies with patients having other variables such as concomitant fractures were excluded. Figure 1 shows the outline of the methodology followed.

## Review

Results

From the initial 4556 hits on performing the search for related publications, we narrowed it down to the most contributing 15 articles. Any conflicts with regards to the studies were clarified by a third author. To define the research question, we used the population, intervention, comparison, outcome (PICO) tool, which is the standard and also endorsed by the Cochrane group [6]. The studies included are enumerated in Table 1.

**Table 1:** Included studies and their characteristics

Author	Year	Number of patients/bone models	Level of evidence
Clinical studies			
Bolhofner et al. [7]	1999	70	III
Alobaid et al. [8]	2004	48	III
Verhofstad and van der Werken [9]	2004	148	III
Laohapoonrungsee et al. [10]	2005	83	III
Malek et al. [11]	2007	389	III
Leung and Tsang [12]	2008	41	III
Riha and Bartonícek [13]	2010	41	III
Baird et al. [14]	2014	327	III
DiPaola et al. [15]	2004	13	III
Fernandes et al. [16]	2018	140	III
Biomechanical studies			
Yian et al. [17]	1997	10 cadaver femurs	III
McLoughlin et al. [18]	2000	8 pairs of cadaver femurs	III
Peleg et al. [19]	2006	4 pairs of cadaver femurs	III
Rog et al. [20]	2017	Synthetic femur models	III
Wang et al. [21]	2020	Synthetic femur models	III

Biomechanical studies

Load to Failure

The two-hole DHS has a statistically significant lesser peak load to failure in a study by Peleg et al. at 3120 Nm compared to 4160 Nm for a four-hole DHS in cadaveric bones [19]. While in another study performed on synthetically produced bones, the difference in peak load to failure was statistically non-significant seen at 644 +/- 106 Nm and 650 +/- 103 Nm for two- and four-hole DHS groups, respectively [20]. McLoughlin et al. too did not find any difference between the findings among the two groups [18].

Differences in the axial and torsional stiffness of the two-plate types are statistically insignificant. They were at 751 +/- 124 N vs 760 +/- 210 N for axial stiffness and 3.58 +/-1.18 N vs 2.95 +/- 0.44 N for torsional stiffness for two- and four-hole DHS, respectively [20].

Fracture Migration on Cyclical Loading

Peleg et al. used three times the average body weight of 70 kg (2100 N) and found the fracture migration to be 33% more in bones with two-hole DHS at 100 loading cycles when compared to a four-hole DHS fixation, which was statistically significant [19]. On the other hand, McLoughlin et al. found it to be similar in both the plates [18].

Stress at Screw Bone Junction

The stress levels at screws themselves within the construct were found to be the maximum in the distal-most screw. In a two-hole DHS, the stress at the distal-most screw was 109-163 Mpa which was more than the yield stress of the bone, indicating that the bone might break at the distal screw in normal cyclic loading. A four-hole plate had distal-most screw stress of 68-91.6 Mpa which was within the safe limits of the human bone [19]. The stress at the distal-most screw in a two-hole plate was found to be significantly more than the four-hole DHS plate screw. This was corroborated by Wang et al. in their biomechanical study where they found the stress at the distal hole in two-hole DHS to be significantly higher at 71.934 than the distal-most screw in a four-hole DHS at 34.684 Mpa [21].

Optimal Side Plate Lengths

Only one study has commented on the optimal length of the DHS plate and it was found to be three holes [17]. They used a 1333.4 Nm vertical loading and found that a third hole substantially protects the previous two holes, and should be used. They further mentioned that the fourth hole did not contribute much to off-loading the stress and can be avoided.

Clinical studies

Operative Duration

The average operative duration for a two-hole DHS was 30.44 min and that for a four-hole DHS was 51.45 min [14]. Another study noted the average duration to be 29 min and 70 min for a two-hole and four-hole DHS, respectively [8]. Other researchers saw the operative times of two-hole DHS in isolation and found them on an average to be 31 min [7], 28 min [13], and 61 min [15].

Blood Loss 

Two studies had similar results with the change in blood haemoglobin (Hb) concentration postoperatively which were statistically non-significant. The study by Barid et al. showed the Hb difference to be 26 g/L for a two-hole DHS compared to 31.3 g/L for a four-hole DHS [14]. Corroborating the same was 22.3 g/L and 29.3 g/L, respectively, in another study [8].

It has to be noted that 43% of the two-hole DHS patients required blood transfusion later in the postoperative period compared to 60% of the four-hole DHS patients, which is statistically significant [14]. This was corroborated by Dipaola et al. who found the transfusion needs to be in 38.5% of the two-hole DHS patients [15].

Radiation Exposure

In a study, the radiation exposure for two-hole DHS was calculated to be average 0.55 min in 41 cases [13]. It was less when compared to the 0.7 min of radiation exposure for a four-hole DHS in another study where they studied 389 DHS cases [11]. There was no direct comparison of the two in any study.

Analgesic Consumption

A shorter incision length used for a two-hole DHS has the theoretical advantage of lesser tissue damage. This has been shown in terms of statistically lower analgesic consumption by the patients in terms of paracetamol intake. For a smaller incision, the consumption was 1.9 g orally averaged over seven days compared to 5.4 g in the traditional incision group [8]. Leung et al. demonstrated similar results with the smaller incision group requiring an average of four tablets of paracetamol over 48 hours compared to eight tablets in the other group, which was again significant [12].

When it came to the stronger analgesics like the opioids there was no statistical difference between the two groups with a smaller incision requiring 169 g of codeine compared to 209 g for the conventional one. It was a similar case with morphine with the value of 15.1 mg vs 25.2 mg for smaller and longer incisions, respectively [8]. The visual analogue scale (VAS) scores were not significantly different either between the two incision lengths [8].

Hospital Stay

Duration of stay analysis demonstrated a non-significant difference in one study with the four-hole DHS had a lower stay duration of 16 days compared to 19 days for a two-hole plate [14]. This was measured from the admission day to the time patient was returned back to pre-injury level of independence in the hospital by physiotherapy and ultimately discharged.

Infection Rates

The infection rates were investigated in two studies showing a statistically insignificant difference of 0.6% for a two-hole plate group and 0.3% for the other group [14]. Another author mentions the infection rates to be 2.7% in the two-hole DHS plate in isolation without any comparison to the two-hole plate [9].

Healing time

The average fracture union duration for a two-hole DHS was found to be around 10 weeks (range: 6-16 weeks) [13] while around average 15 weeks by others [7,10]. There was no direct comparison of the two groups in any of the studies.

Failure Rates

A statistically significant difference was seen in unstable intertrochanteric fractures with a failure value at 24.4% for the two-hole group and 10.8% for the four-hole plate group. The failure rates were statistically insignificant in stable fractures at 6.3% and 4.9% for the two- and four-hole groups, respectively [14]. In a 140-patient series, Fernandes et al. found the implant failure to be statistically insignificant at 13.9% for four-hole plates and 12.5% for two-hole plates, but the study as others was not randomised [16]. A few other researchers obtained failure rates in two-hole DHS in isolation and found them to be 4.8% [10], 2.1% [9], 3.1% [13], and 4.5% [7].

Discussion

For stable intertrochanteric fractures of the femur, the DHS has been the gold-standard implant for fracture fixation. There have been a few instances of a two-hole DHS being used but generally, the four-hole plate is the one preferred by most and the discussion on the length of side plate has had only limited discussion in previous studies. Surgeons require compelling and reliable evidence to change their practice and use a two-hole DHS without any reservations. With our study, we aimed to as far as possible address this question, compile the evidence base, and give the readers a more comprehensive view. At the same time, PRISMA guidelines were adhered to while performing this systematic review to keep the evidence obtained as valid as possible [22].

Biomechanical Parameters

The most important parameter of load to failure demonstrated the two-hole DHS to have statistically significant lower values in one of the studies done on four cadaveric bones [19]. This theoretically indicates that the two-hole DHS should fail before a four-hole one on loading, in identical fractures. Though, it was not always the case as seen in a few other studies [18,20]. Similarly, stress levels at the distal-most screw were statistically higher in two-hole plates pointing towards a possible fracture at the screw bone junction at that screw.

Cyclical loading mimicking everyday load-bearing showed that the two-hole DHS had significantly more fracture fragment migration when cyclically loaded multiple times with three times body weight. Moreover, a higher stress level was seen at the distal screw in the two-hole plate [19]. It points towards poorer biomechanical outcomes and higher failure rates in identical fractures, more so in high BMI individuals. Hence keeping the weight of the patient in mind while deciding the implant becomes important as well. It must be noted that how this would behave in a clinical setting with this theoretical biomechanical disadvantage can only be established with a future randomised control trial. While interestingly the property of axial and torsional stiffness was studied and was found to be similar in both the implants [20].

Clinical Parameters

It is well known that a prolonged operative time has been linked to a worse outcome in surgical patients [23]. Precisely, which is a significant factor considered by quite a few studies. Two-hole DHS has been found to have a lower operating time which correlated well with the reduced operating steps. Moreover, some studies showed a longer operative duration for four-hole DHS, with one study quoting a difference as high as 31 min [8]. Interestingly, one study has shown the time required for a four-hole DHS to be similar to a two-hole one [15]. The studies do not mention from which-to-which point the time measurements are being done and since it is not under strict control, it can be quite subjective.

It needs to be realised that since these studies were not a randomised control trial, a four-hole plate would most likely have been used for a more complicated and unstable fracture, naturally requiring more time to fix at every step of the surgery.

None of the studies demonstrated any statistically significant higher loss of hemoglobin postoperatively [8,14] but when the postoperative transfusion rates were compared, one study showed a significantly higher rate of transfusion in a four-hole DHS [14]. It does appear to be happening due to the first major perforator from the femoral artery being at 9.3 cm from the trochanteric flare, which invariably gets cut or avulsed in a four-hole plate use [8]. Interestingly, there was no mention of increased mortality or morbidity with the four-hole plate despite a higher transfusion frequency. Hence, clinically both the plates do appear to be similar in this regard.

An infrequently compared variable, radiation exposure was also included in our analysis. From a couple of studies, it is evident that while performing a four-hole DHS, an average of nine seconds (0.15 min) difference in radiation exposure was found [11,13]. Incidentally, it is seen that the radiation exposure varies greatly with the experience of the surgeon in a DHS surgery, which can very well be the dominant factor rather than the size of the side plate [24].

Keeping the pain under control is one of the challenges in a ward after surgery and is quantified by analgesic consumption. Paracetamol use was significantly lower in two-hole DHS in two of the studies while the use of stronger painkillers like opioids did not have any significant difference in consumption rates over seven days postoperatively [8,12]. Corroborating analgesic use, the VAS scores were not significantly different either [8]. Hence, in terms of postoperative pain, the two plate types do not differ. A four-hole DHS has a statistically insignificant shorter hospital stay which has been attributed by the studies to be influenced by the preoperative status and comorbidities of the patient, rather than the surgery itself [9,14]. While the absolute numbers would not appear very drastically different but a shorter stay even by a day has been linked to lesser complications in the patients [25].

A lot of researchers have looked at the healing time of two and four-hole DHS and found them to be very similar in their studies [7,10,13]. Infection rates were similar for the surgeries performed with either of the plates in similar fracture patterns [9]. Failure rates were higher in the two-hole plate group but statistically non-significant in stable fractures [14]. Though again it must be emphasised that the studies were not randomised control trials and the two-hole DHS could have been used in undisplaced fractures predominantly.

Limitations

None of the studies were randomised control trials which keep the level of evidence of the included studies low. In addition, the implant selection was purely based on fracture type and surgeon preference in all the studies. There is no mention of bone quality in any of the papers, which can very well influence the implant selection and outcome parameters. There is a very high possibility that four-hole DHS would have been used for more complex fractures and in patients with poorer bone quality. Hence, it is possible that variables such as operation time, blood loss, and failure rates would have been affected due to the same. Besides, the outcome measures in all the included studies are not exhaustive and leave out important parameters like validated functional outcomes like Harris Hip Score [26]. Another thing to keep in mind is the non-inclusion of the locking DHS plate in the study which probably provides better fixation than non-locking plates, especially in osteoporotic bone.

## Conclusions

Looking at these clinical and biomechanical studies, we can clearly see that the two-hole DHS cannot be wholeheartedly recommended for stable intertrochanteric fractures of the femur, let alone the unstable ones. The two-hole DHS does lag behind significantly in biomechanical parameters such as load to failure, cyclical loading, stresses at distal screw. It is found equivalent in parameters of fracture healing time, infections, radiation exposure, analgesic consumption, hospital stay and failure rates. While there are a few advantages of the two-hole plate in terms of blood loss and operating time, evidently the objective biomechanical studies and higher clinical failure rates, expose the overall weakness of the two-hole DHS very clearly.

With our systematic review, we recommend the use of four-hole DHS for the stable intertrochanteric neck of femur fractures at this point. In addition, there has been no mention of any measured increased mortality or morbidity with a four-hole plate compared to a two-hole plate. To have a strong recommendation and comprehensively answer the question we set out with, a good quality randomised control trial is required which should include all the discussed parameters and a functional hip scoring system in a stable intertrochanteric fracture of neck of femur fixed with two and four-hole DHS.
